# The frequency of non-syndromic distomolar teeth in a Greek population sample?

**DOI:** 10.4317/jced.52411

**Published:** 2015-12-01

**Authors:** Anastasia Mitsea, Emanouel Vardas, Angeliki Papachatzopoulou, Georgios Kalfountzos, Minas Leventis, Kostas Tsiklakis

**Affiliations:** 1 DDS, MSc DMFR, MSc FO, PhD Med, PhD Dent, Lecturer, Department of Oral Diagnosis and Radiology, Dental School University of Athens, Greece; 2 DDS, MSc, MSc, PhD Dent, Clinical Associate, Hospital Dentistry, Dental School, National and Kapodistrian University of Athens, Greece; 3 DDS, MSc Postgraduate student, Department of Oral Diagnosis and Radiology, Dental School University of Athens, Greece; 4 DDS, MSc, PhD Dent, Clinical Associate, Dental School, National and Kapodistrian University of Athens, Greece; 5 DDS, MSc, PhD Dent, Professor and Head, Department of Oral Diagnosis and Radiology, Dental School University of Athens, Greece

## Abstract

**Background:**

To investigate the frequency of non-syndromic distomolars in a Greek population sample.

**Material and Methods:**

The study population of this retrospective study consisted of 859 Orthopantomograms (OPGs) of 425 male and 434 female patients, attended the Department of Oral Diagnosis and Radiology, Dental School of Athens seeking for treatment. The OPGs were taken as a part of the patients treatment planning. Patients’ mean age was 33.57 years. Exclusion criteria from this study was cleft lip ± palate and diseases associated with systemic conditions and syndromes (such as cleidocranial dysplasia and Gardner syndrome). OPGs were only included in the study if at least one 3rd molar was present. The data collected were the number of 3rd molars, the number of distomolars, the age and the gender of each patient, information concerning previous extraction of 3rd molars. Statistical evaluation of the data included descriptive and bivariate analyses (Chi-square test and Spearman’s rho correlation coefficient). In an attempt to further estimate the correlation between the presence of upper and lower 3rd conditions we assumed that the absence of 3rd molars, the presence of 3rd molars, and the presence of distomolars was ordinal in nature and we calculated the Spearman Correlation Coefficient.

**Results:**

The number of distomolars was greater in the maxilla than in the mandible. In the maxilla the distomolars were located almost equally in both left and right side. It was more possible lower left distomolars to be present in males than in females. Furthermore, males present higher prevalence of supernumerary teeth than females.

**Conclusions:**

Early radiographic diagnosis of distomolars is fundamental so as to prevent complications such malocclusion, delayed eruption or displacement root or/ and resorption of adjacent teeth, pulp necrosis, follicular cyst, pain.

** Key words:**Non syndromic, distomolars, supernumerary molars, fourth molars.

## Introduction

Dental radiographs and especially new imaging techniques such as digital radiographs and Cone Beam Computed Tomography (CBCT) enable us to easily detect numerous incidental findings such as supernumerary impacted teeth or teeth abnormalities.

**Figure 1:**
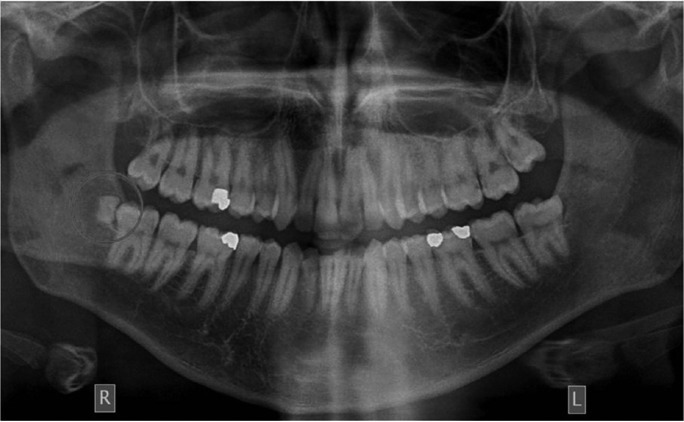
A distomolar located in the lower right area.

Supernumerary teeth may present both in primary and permanent dentition. In particular, their prevalence is 5 times lower in the primary dentition (ranging from 0.3% to 0.8% in the Caucasian population) than in the permanent dentition (ranging from 0.5% to 5.3%) and presents geographic variation (1-5). They might be single or multiple and occur more often in males than in females. Furthermore, these teeth present a delayed development in relation to normal teeth, could be located in one or in both jaws, (although they present a predilection in the maxilla), may be erupted or impacted, symptomatic or asymptomatic, located unilaterally or bilaterally (1-6). Supernumerary teeth may occur as a clinical finding in syndromes such as cleidocranial dysplasia, in cleft lip and palate patients, otherwise they might be a non-syndromic finding (2,5). According to previous reports 76% to 86% of non syndromic cases present only one supernumerary tooth (Fig. 1), 12% to 23% present two supernumerary teeth and only 1% present multiple supernumerary teeth (6-8). Supernumerary teeth are classified according to their location in the dental arches, or to their morphology (1-3,5). Infrequently they are sited in molars region and categorized in paramolars and distomolars. Specifically, paramolars usually are rudimentary teeth located on the molars region while distomolars are located distally to the third molars (1,5).

If distomolars are impacted they may cause complications such as impaction, root resorption or pulp necrosis of the adjacent tooth, pain in the molar area, infection, formation of diastema, follicular cyst, neoplasms and neuralgias of the trigeminous nerve. In case that they erupt they may cause malocclusion, retention or ectopic eruption, delayed eruption of the adjacent teeth, mandibular disorders, periodontal disease and caries (2,6,8).

New imaging techniques such as CBCT enable us to accurately evaluate the intraosseous location, inclination and morphology of impacted supernumerary teeth. Additionally, it is possible to assess their relation to superimposed adjusted teeth or anatomical structures such as maxillary sinus and nasal cavity. Early detection of supernumerary teeth is important for early intervention that enhances the therapeutic result (9-12).

The aim of this study is to estimate the frequency of non-syndromic distomolars in a Greek population sample.

## Material and Methods

In this retrospective study conducted in the Department of Oral Diagnosis and Radiology of Dental School University of Athens. Initially we examined 1011 Orthopantomograms (OPGs) from the database of the Department of Oral Diagnosis and Radiology, Dental School of Athens between the years 2011 and 2013 seeking for treatment. The OPGs were taken as a part of the patients treatment planning and not for the purpose of this study. The protocol of our study has been approved by the Ethics and Research Committee of the University of Athens (Reference Number 267A). A distomolar tooth was defined as a tooth located distally to the third molar, as an accessory fourth molar (1).

From each patient’s dental records the following data were collected: the age, the gender and information concerning previous extraction of 3rd molars. Patients with history of extractions of 3rd molars or supernumerary teeth or with any disease affecting the normal development of the permanent dentition any inherited diseases or syndromes such as cleidocranial dysostosis, Down’s or Gardner syndrome were excluded from this study. The inclusion criteria were, that belonged to Greek Caucasian subjects that had not undergone any extractions in the third molar regions with at least one 3rd molar present.

Eventually, only 859 OPG’s included in this study following the above inclusion and exclusion criteria. All radiographs were studied by 3 dentomaxillofacial radiologists, under the same conditions and collected the number of distomolars per patient and their location.

Statistical evaluation of the data included descriptive and bivariate analyses (Chi-square test and Spearman’s rho correlation coefficient). Statistical evaluation of the two way frequency tables was performed using the Chi square statistic. In order to further estimate the magnitude of the correlation between the presence of upper right 3rd molar, the lower right 3rd molar, the upper right and the lower right distomolars as well as the upper left 3rd molar, the lower left 3rd molar, the upper right distomolars and the lower left distomolars we chose ordinal regression. Ordinal regression captures correlations between all possible pairs of groups concerning the presence or not of 3rd molar and distomolars. In this regression we assumed that the absence of 3rd molars, the presence of 3rd molars, and the presence of distomolars was ordinal in nature, i.e. absence of 3rd molar=0, presence of 3rd molar=1, and presence of distomolar =2, and we calculated the Spearman Correlation Coefficient. All statistical indices were read for 0.05% statistical significance. Analyses were performed using the STATISTICA 10.0 for Windows software (StatSoft inc.).

## Results

Of the 859 Orthopantomograms (OPGs) 425 (49.48%) belonged to males and 434 (50.52%) to females patients. The age range of the sample was 12 to 80 years and the mean age was 33.57 (SD 14.75) years. The mean age of male patients was 34.45 years (SD 14.64) and the mean age of the female patients was 32.78 years (SD 14.83). There was a negative correlation between age and the presence of distomolars in a statistically significant level (*p*<0.05).

Distomolars prevalence was very low range from 0.95% to 0.11% in both jaws (*p*<0.05) as a total of 20 distomolars was found. More distomolars 0.89% (N=17) were located in the maxilla than in the mandible 0.16% (N=3) in a statistically significant level (*p*<0.05). In both jaws the distomolars were located almost equally in both left and right side (*p*<0.05). Distomolar in the lower left area had the lowest frequency (approx. 0%) (*p*<0.05) ( Table 1).

**Table 1:**
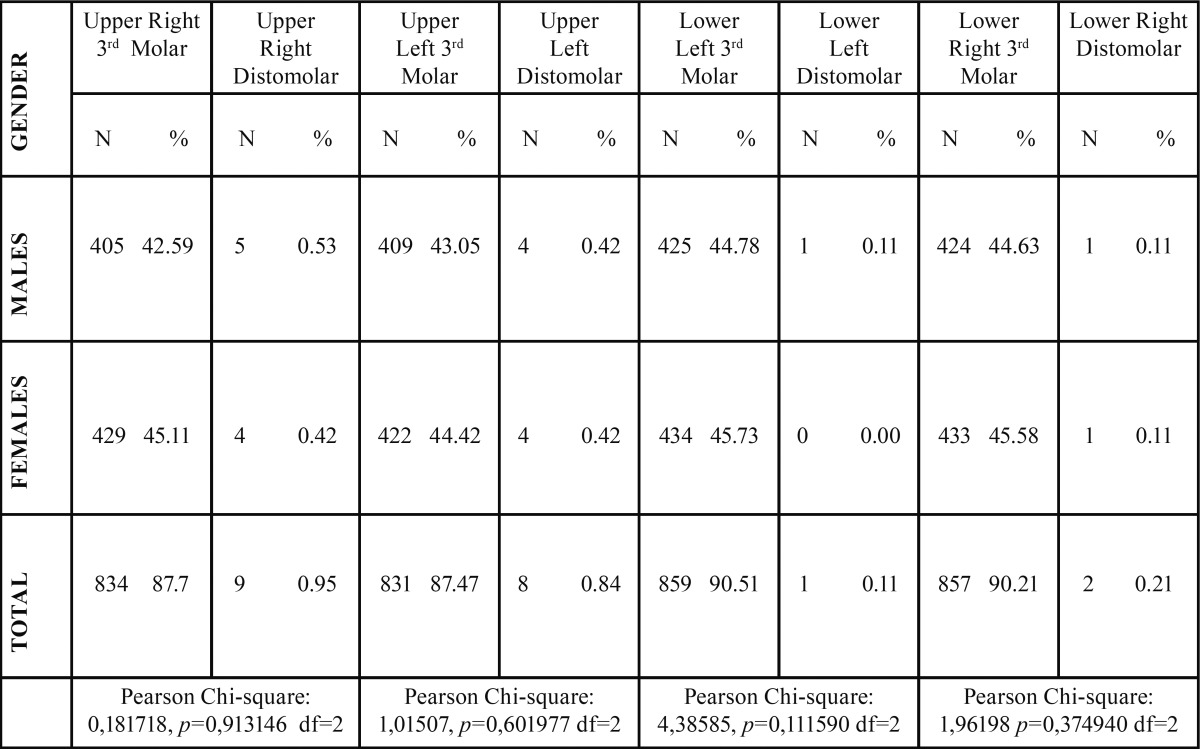
The prevalence of distomolars between genders.

The prevalence of distomolar teeth in males was 0,29% (N=11) and in females 0,23% (N=9). Also, males tended to more possibly have lower left distomolar compared to females ( Table 1).

As it can be seen in  Table 2 in the majority of cases the presence of lower right 3rd molar corresponded highly with the presence of upper right 3rd molar and upper right distomolar. More specifically the 82.19% in the entire sample and 82.72% and 80.04% in males and females respectively for lower right 3rd molars and 0.90% in the entire sample and 1.08% in males and 0.82% in females concerning the upper right distomolars. These findings were statistically significant. The Spearman Rank R ranged between 0.21 for females and 0.30 for males, with the entire sample having an R of 0.25 suggesting a weak but statistically significant correlation between lower right 3rd molars and the presence of upper right distomolars. The correlation was higher in males as compared to the females ( Table 2).

**Table 2:**
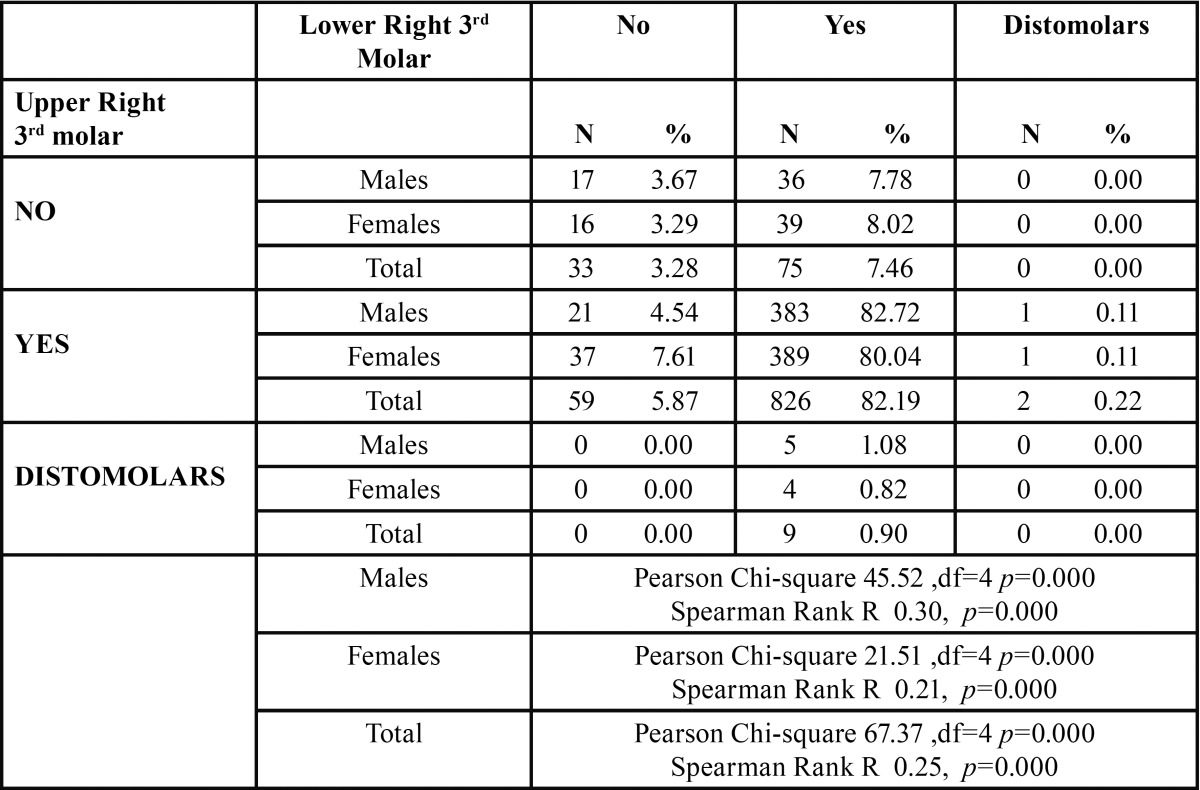
The prevalence of distomolars in the right side.

As it can be seen in  Table 3 in the majority of cases the presence of lower left 3rd molar corresponded highly with the presence of upper left 3rd molar and upper left distomolar. More specifically 83.68% in the entire sample and 86.12% and 79.71% in males and females respectively concerning the upper left 3rd molar and 0.70% in the entire sample and 0.65% and 0.82% in males and females respectively concerning the upper left distomolars. These findings were statistically significant. The Spearman Rank R ranged between 0.27 for females and 0.47 for males with the entire sample having an R of 0.35 suggesting a weak to moderate, statistically significant correlation between lower left 3rd molars and the presence of upper left distomolars. The correlation was higher in males as compared to the females ( Table 3).

**Table 3:**
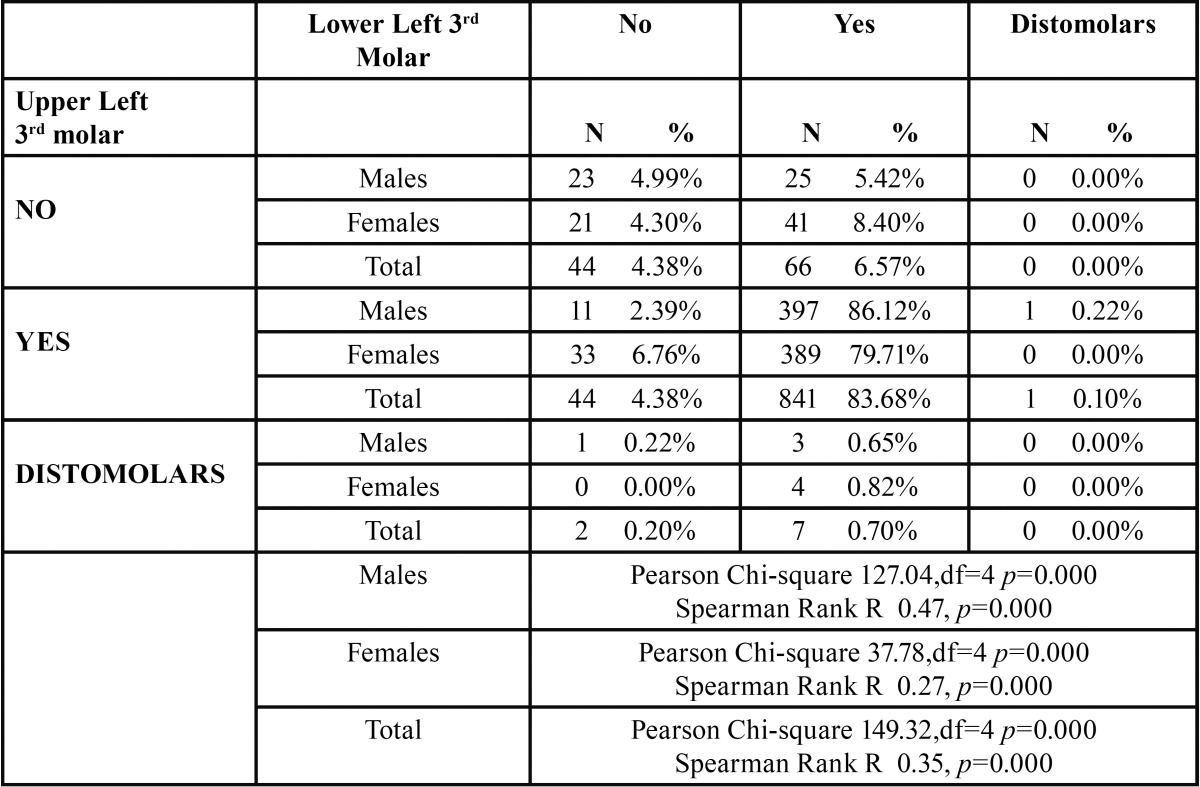
The prevalence of distomolars in the left side.

## Discussion

Although a number of theories have been developed, the etiology of hyperdontia is still blurred. The most accepted theory is the suggestion of a pattern of multifactorial inheritance originating from local, independent, conditioned hyperactivity of the dental lamina (3).

**Figure 2:**
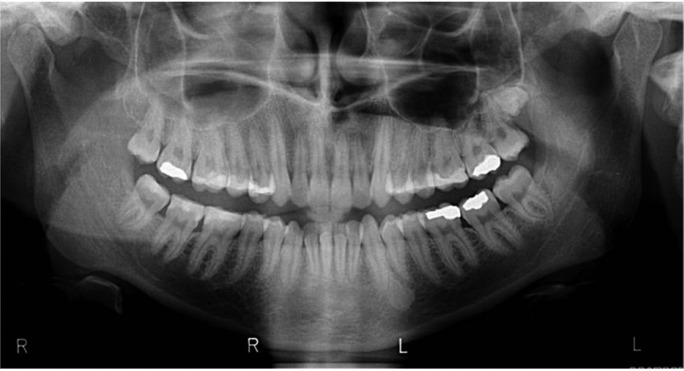
An impacted distomolar located in the upper left area.

Most frequently supernumerary teeth are located in the maxilla rather than in the mandible (Fig. 2). They are classified according to their morphology to conical, tuberculate, supplemental, and odontoma types (1,4). The classification according to the location includes mesiodens, paramolars and distomolars. A conical tooth located between the incisors called mesioden. If located buccally or lingually of the first, second or third molars called paramolar and if located distally to the third molars called distomolar. Distomolars are smaller than normal second and third molars (1,3,4).

According to Shapira and Kuftinec the order of decreasing frequency is: upper central incisors, molars (especially upper molars), premolars, followed by lateral incisors and canines (13). Supernumerary teeth are rare in the lower incisors area and in the canine area (3).

Several studies have been performed concerning the frequency of distomolars in different population and ethnic samples. Fourth, fifth, sixth, and even seventh molars have been observed although fourth molars are seen most frequently (14). Kara *et al.* reported that the prevalence of fourth molars was 0.33% (15), Cassetta *et al.* found it to be 0.18% (16) and Kaya *et al.* resulted a percentage of 0.26% (17).

The gender distribution of these teeth shows males to be more affected in the permanent dentition (1,4,7,17). This result comes with an agreement with the findings of our study. Additionally we resulted that a larger proportion of distomolars was found in the maxilla than in the mandible. This is also in accordance with the findings of other studies (1,16-19).

Generally a supernumerary tooth may cause a number of complications such malalignment of the dentition, eruption disturbances in the adjacent teeth such as crowding, delayed eruption, displacement, rotation, root resorption and pulp necrosis of adjacent teeth. Moreover periodontal disease, increased incidence of dental caries in adjacent teeth, abnormal diastema or premature space closure, cystic lesions, and, pain may occur (2,3,20,21).

Usually supernumerary teeth may remain impacted or present a delay formation and eruption. Moreover, in case that, they erupt they may erupt inverted, in an ectopic or abnormal position (1). Thus, detailed clinical and radiographic evaluation is a fundamental for the early diagnosis, proper evaluation, appropriate treatment and redaction of complications in patients who present distomolars. Therefore, epidemiological studies related to supernumerary teeth can be useful to clinicians (16,20,22). Depending on the location, different dental radiographic techniques may be used for imaging the exact positioning of the supernumerary teeth (3). Treatment procedures are individualized taking into account the risk and benefit ratio, depending on the type and position of the supernumerary tooth and the associated effects and symptoms to the adjacent teeth (19). In some cases, is preferable to monitor a supernumerary tooth rather than to remove it (3). Cone Beam Computed Tomography (CBCT) is indicated when extraction of supernumerary teeth is decided. Furthermore, in any case of surgical removal or monitoring of distomolars, CBCT is a valuable tool to easily detect the accurate location of these teeth and their relation with adjacent anatomical structures (23).

In conclusion, distomolars frequency ranged from 0.84% to 0.95% in the maxilla and from 0.11 to 0.22% in the mandible in this population sample. Moreover distomolars presented a clear male predilection in this sample and were most frequently located in the maxilla in both genders. Distomolars occurred more frequently in the right side than in the left. A positive and significant correlation was found between all 3rd molars and the distomolars of the upper jaw in this study. Also, age presented a negative and statistically significant correlation with all 3rd molars, probably indicating the increase of extraction rate of these molars with the increase of age. These findings come to an agreement with the results of studies in different population and ethnic samples.
